# Camptothecin targets WRN protein: mechanism and relevance in clinical breast cancer

**DOI:** 10.18632/oncotarget.7906

**Published:** 2016-03-03

**Authors:** Raghavendra A. Shamanna, Huiming Lu, Deborah L. Croteau, Arvind Arora, Devika Agarwal, Graham Ball, Mohammed A. Aleskandarany, Ian O. Ellis, Yves Pommier, Srinivasan Madhusudan, Vilhelm A. Bohr

**Affiliations:** ^1^ Laboratory of Molecular Gerontology, Biomedical Research Center, National Institute on Aging, NIH, Baltimore, Maryland, USA; ^2^ Academic Unit of Oncology, Division of Cancer and Stem Cells, School of Medicine, University of Nottingham, Nottingham NG51PB, UK; ^3^ School of Science and Technology, Nottingham Trent University, Clifton Campus, Nottingham NG11 8NS, UK; ^4^ Developmental Therapeutics Branch and Laboratory of Molecular Pharmacology, National Cancer Institute, Bethesda, Maryland, USA

**Keywords:** Werner syndrome, topoisomerase I, senescence, protein degradation, DNA damage response, breast cancer

## Abstract

Werner syndrome protein (WRN) is a RecQ helicase that participates in DNA repair, genome stability and cellular senescence. The five human RecQ helicases, RECQL1, Bloom, WRN, RECQL4 and RECQL5 play critical roles in DNA repair and cell survival after treatment with the anticancer drug camptothecin (CPT). CPT derivatives are widely used in cancer chemotherapy to inhibit topoisomerase I and generate DNA double-strand breaks during replication. Here we studied the effects of CPT on the stability and expression dynamics of human RecQ helicases. In the cells treated with CPT, we observed distinct effects on WRN compared to other human RecQ helicases. CPT altered the cellular localization of WRN and induced its degradation by a ubiquitin-mediated proteasome pathway. WRN knockdown cells as well as CPT treated cells became senescent and stained positive for senescence-associated β-galactosidase at a higher frequency compared to control cells. However, the senescent phenotype was attenuated by ectopic expression of WRN suggesting functional implication of WRN degradation in CPT treated cells. Approximately 5-23% of breast cancer tumors are known to respond to CPT-based chemotherapy. Interestingly, we found that the extent of CPT-induced WRN degradation correlates with increasing sensitivity of breast cancer cells to CPT. The abundance of WRN decreased in CPT-treated sensitive cells; however, WRN remained relatively stable in CPT-resistant breast cancer cells. In a large clinical cohort of breast cancer patients, we find that WRN and topoisomerase I expression correlate with an aggressive tumor phenotype and poor prognosis. Our novel observations suggest that WRN abundance along with CPT-induced degradation could be a promising strategy for personalizing CPT-based cancer chemotherapeutic regimens.

## INTRODUCTION

The RecQ family of helicases contains highly conserved and ubiquitously expressed proteins that unwind DNA in the context of replication, repair, transcription, chromatin remodeling and telomere maintenance [1, 2]. Human and most mammalian cells encode five RecQ-like (RECQL) helicases: RECQL1, Bloom (BLM), Werner (WRN), RECQL4 and RECQL5. These helicases display unique as well as overlapping functions in DNA metabolism. They bind specific DNA structures and catalyze unwinding and annealing of DNA strands to resolve DNA replication forks, D-loops, G-quadruplex structures and Holliday junctions [1, 2]. Mutations in *BLM, WRN* and *RECQL4* are associated with autosomal recessive diseases. Loss of function of BLM and WRN is associated with Bloom syndrome (BS) and Werner syndrome (WS) respectively, while RECQL4 is associated with Rothmund-Thomson (RTS), RAPADILINO and Baller-Gerold (BGS) syndromes[1-3].

In general, cells with defects in DNA repair have increased risk of transformation to a pre-cancer or cancer phenotype. WS and BS patients exhibit increased incidence of cancer. The most common neoplasias in WS patients are thyroid cancer, malignant melanoma, meningioma, soft tissue sarcoma, osteosarcoma, breast cancer and leukemias [3, 4]. Increased WRN expression is observed in several cancer cell lines and depletion of WRN induces cell death in these cells [5]. Irinotecan treatment enhanced the survival of colorectal cancer patients who expressed lower WRN [6].

The plant alkaloid camptothecin (CPT) and its derivatives, irinotecan and topotecan, represent an important class of drugs used in chemotherapy. These drugs specifically target DNA topoisomerase I (Top1), an enzyme that transiently creates DNA single-strand breaks to reduce supercoiling during replication and transcription [7, 8]. CPT generates cytotoxic covalent reaction intermediates, CPT-DNA-Top1, by inhibiting the re-ligation step of the Top1 catalytic cycle. The cytotoxic effect of the CPT-DNA-Top1 intermediate is S-phase-specific, and is thought to reflect collision events between the replication machinery and the cytotoxic lesion [7, 8]. When cells accumulate many CPT-DNA-Top1 lesions, the DNA damage response (DDR) and associated pathways are activated [8]. Subsequent to DDR activation, DNA repair factors, including RecQ helicases are recruited to the DNA lesions and/or to stalled DNA replication forks. All human RecQ helicases are important for cell survival after CPT treatment [9-13]. WS and BS patient cells are hypersensitive to inhibitors of Top1 and DNA interstrand crosslinking agents, and a synergistic increase in chromosomal aberrations is observed in BLM-WRN double knockout cells exposed to these agents [11]. RECQL4-deficient RTS patient cells and RECQL1 and RECQL5 knockdown cells are also sensitive to CPT [9, 12, 13]. However, studies identifying the mechanisms by which CPT or its analogs exert their effects on human RecQ helicases are limited. In this study, we tested the effects of CPT on the five RecQ helicases in cellular studies and bioinformatically analyzed the association between CPT sensitivity and WRN gene expression. Further we analyzed the expression profiles of WRN and Top1 in a large cohort of human breast cancers to identify any correlations between gene expression and breast cancer specific survival. This study spans from biochemical and cellular work through bioinformatics to a clinical study.

CPT treatment specifically altered the stability and subcellular localization of WRN, while similar effects on other RecQ helicases were not observed. In CPT-treated cells, a large fraction of WRN re-localized to the cytoplasm and was selectively degraded by the ubiquitin proteasome pathway. CPT-induced WRN degradation was independent of p53 status, and the extent of degradation was associated with the sensitivity of the tumor cells to the anticancer drug. WRN degradation was more extensive in CPT-sensitive breast cancer cells than in CPT-resistant cells. However, CPT-dependent degradation of Top1 was extensive in all cell lines tested. In the METABRIC (Molecular Taxonomy of Breast Cancer International Consortium) cohort comprising 1977 breast cancers, ~20% of tumors were found to express high Top1 mRNA and ~83% were found to express high WRN mRNA. Altered Top1 and WRN expression was not only associated with aggressive breast cancers but also correlated with adverse prognostic outcome in patients. Interestingly, in patients with estrogen receptor (ER)-positive breast cancers, high WRN and high Top1 levels were associated with a bad prognosis. Together these results suggest that WRN, but not the other RecQs, is a target of CPT in mediating chemotherapeutic effects in the tumor cells.

## RESULTS

### Stability, expression and subcellular distribution of WRN in CPT-treated cells

CPT and its derivatives specifically target Top1, and the five human RecQ helicases play essential roles in cell survival after CPT treatment [9-13], thus we tested the effects of CPT on the expression dynamics of all five human RecQ helicases to identify unique or shared responses to CPT treatment.

**Figure 1:**
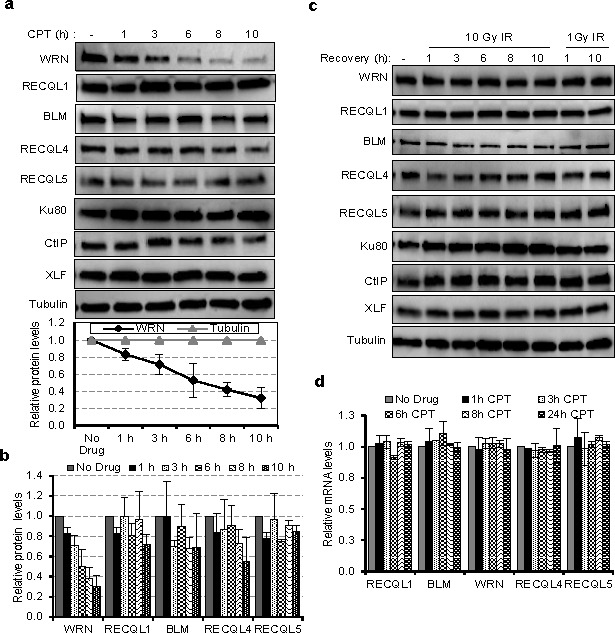
Expression of RecQ helicases in CPT and IR treated cells **a.** Half-life of DNA repair proteins in CPT treated cells. Lysates from U2OS cells were treated with 10 μM CPT for the indicated times and immunoblotted for DNA repair proteins viz., Ku70, CtIP, XLF and the RecQ helicases WRN, BLM, RECQL1, RECQL4 and RECQL5. Below; graph showing the relative levels of WRN protein normalized to tubulin from three independent experiments. **b.** Bar graph showing the protein levels of RecQ helicases in CPT treated cells. **c.** Immunoblots showing the expression of DNA repair proteins as in panel A from IR treated U2OS cells. **d.** Quantitative RT-PCR analysis showing mRNA levels of *RecQ* genes in CPT treated U2OS cells. Expression of *GAPDH* mRNA was used for normalization. Error bars represent standard deviation from two independent experiments.

U2OS cells were treated with 10 μM CPT for 1, 2, 6, 8 or 10 hours, and lysed with IP lysis buffer [14]. Proteins in the supernatant fraction, obtained after centrifugation, were analyzed by immunoblotting for WRN, BLM, RECQL1, RECQL4, RECQL5, Ku80, CtIP and XLF (Figure 1a). Exposure to CPT caused a decrease in the abundance of WRN protein, but not of the other proteins tested (Figure 1a and 1b). Quantification of the immunoblots indicated that WRN protein decreased significantly in a time-dependent manner from ~1 to 10 h after treatment with CPT (Figure 1a graph). This reduction was not observed for BLM, RECQL1, RECQL4 and RECQL5 or for the DNA repair proteins Ku80, CtIP, and XLF (Figure 1a and 1b). The half-life of WRN protein is reported to be approximately 6 h [15], consistent with its apparent half-life in this study (Figure 1a graph). Interestingly, cells treated with 1 or 10 Gy ionizing radiation (IR) did not show a decrease in WRN abundance at time points up to 10 h after irradiation (Figure 1c). These results indicate that the effect of CPT on WRN protein levels could be specific.

The mRNA transcripts encoding all five RecQ helicases were also quantified in CPT-treated and untreated cells (Figure 1d). For all RecQ helicases, transcript abundance was unaffected up to 24 h with CPT treatment. This indicates that CPT induces (Figure 1a) specific post-translational degradation of WRN protein, or that it destabilizes WRN protein by another mechanism.

**Figure 2:**
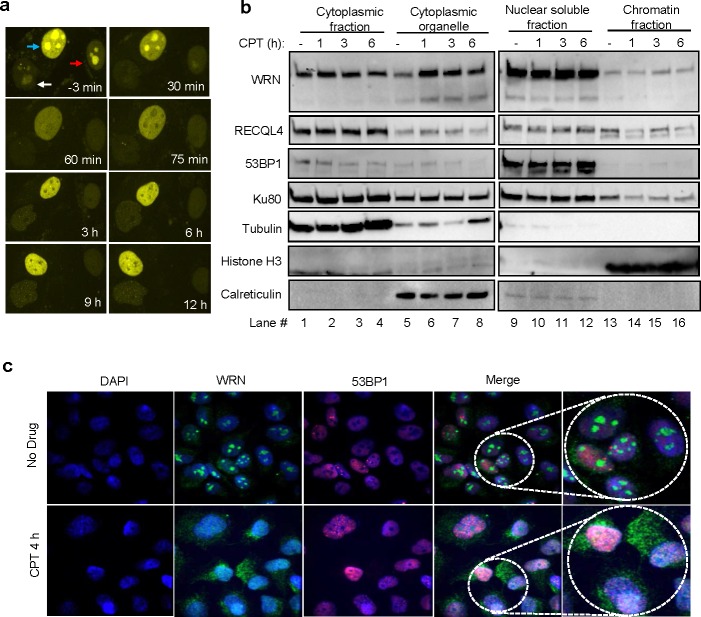
CPT-induced re-localization of WRN **a.** Dynamic distribution of YFP-WRN in CPT-treated cells. Live cell time-lapse micrographs from CPT (10 μM) treated U2OS cells stably expressing YFP-WRN. Arrows point the nucleus of YFP-WRN expressing cells. **b.** WRN distribution in the subcellular compartments. Immunoblots indicating the relative levels of WRN in the cytoplasmic, cytoplasmic organelle, nuclear soluble and chromatin fractions of U2OS cells treated with and without CPT. Time indicate the length of the exposure to CPT. **c.** Enrichment of endogenous WRN in the cytoplasm. Confocal microphotographs showing WRN and 53BP1 localization in U2OS cells treated with CPT. Representative images from two independent experiments. Microscope images were taken with 40X objective.

WRN protein distribution was analyzed in U2OS cells stably expressing YFP-WRN and treated with CPT using confocal microscopy (Figure 2a). Live-cell microscopy results indicated that YFP-WRN localized primarily to the nucleolus, re-localized to the nucleoplasm ~60 min after exposure to CPT, after which YFP-WRN fluorescence declined to a low level in the nucleolus (Figure 2a). YFP-WRN foci were also observed in the nucleoplasm of CPT-treated cells, suggesting recruitment of WRN to CPT-induced DNA damage. To examine the subcellular distribution of WRN, proteins from the cytoplasm, cytoplasmic organelles, nuclear-soluble and chromatin fractions were isolated and analyzed by Western blotting (Figure 2b). The results suggest that WRN is enriched in cytoplasmic organelles (Figure 2b, compare lane 5 with 6-8) after CPT treatment. Analysis of subcellular protein fractions from IR treated cells did not show significant changes in the distribution pattern of WRN compared to untreated cells (Supplementary Figure S1a). Further, results from immunostained cells indicated that endogenous WRN resides in the nucleolus of cells (Figure 2c top panel and [16]) and that, upon treatment with CPT, re-localizes to the nucleoplasm and cytoplasm (Figure 2c bottom panels). To test the role of translation in CPT-dependent enrichment of WRN in the cytoplasmic organelle fraction observed in Figure 2b, cells were treated with cycloheximide (CHX) along with CPT. Inhibition of translation did not affect CPT-induced re-localization of WRN (Supplementary Figure S1b), suggesting that the WRN enrichment is not dependent on new protein synthesis. Collectively these results demonstrate that WRN undergoes re-localization in cancer cells treated with CPT.

### CPT-induced ubiquitination and degradation of WRN

**Figure 3:**
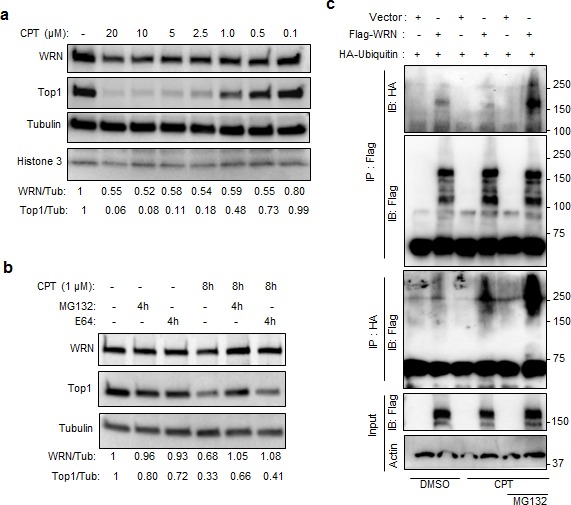
Ubiquitin proteasome-mediated degradation of WRN in CPT treated cells **a.** CPT-induced WRN down regulation is associated with Top1 degradation. Immunoblots showing WRN and Top1 protein levels in U2OS cells treated with indicated concentration of CPT for 16 h. **b.** Proteasome inhibitor MG132 inhibits the effect of CPT on WRN and Top1. U2OS cells were treated with 10 μM MG132 to inhibit proteasome pathway and with 10 μM E64 to inhibit lysosome pathway. **c.** Ubiquitination of WRN in CPT treated cells. Immunoblots showing ubiquitinated (HA-ubiqutin) 3xFlag-WRN in immuno­precipitated fractions of CPT (1μM) treated 293T cells expressing 3xFlag-WRN and HA-ubiqutin.

To identify the CPT concentrations that induce WRN degradation, U2OS cells were treated with 0.01, 0.05, 0.1, 0.5, 1, 5, 10 and 20 μM CPT for 16h and extracts were prepared by lysing the cells with RIPA buffer followed by brief sonication. Our results showed that the WRN protein is downregulated in cells exposed to 0.5 to 20 μM CPT, conditions that induce degradation of Top1 (Figure 3a and Supplementary Figure S2a). We previously reported that WRN interacts with Top1 [17] and other studies have shown that CPT-induced degradation of Top1 occurs in an ubiquitin- and proteasome-dependent manner [18, 19]. Therefore, it seemed possible that WRN downregulation might be ubiquitin- and proteasome-dependent. Consistent with this possibility, the proteasome inhibitor MG132 suppressed CPT-induced degradation of WRN and Top1 in cells treated with 1 μM CPT (Figure 3b and Supplementary Figure S2b). Further, when 293T cells co-expressing 3xFlag-WRN and HA-ubiquitin were treated with CPT and MG132 (Figure 3c), and cell lysates were analyzed after immunoprecipitation, ubiquitin and WRN co-immunoprecipitated with HA or FLAG antibodies. MG132 stabilized HA-ubiquitinated Flag-WRN, implicating proteasome-mediated degradation of WRN in CPT-treated cells (Figure 3c). We found no evidence that WRN was enriched in lysosomes, using cells in which the lysosome was fluorescence-labeled with lysosome-specific GFP (Supplementary Figure S3). These results suggest that CPT-stimulated degradation of WRN is lysosome-independent, but ubiquitin- and proteasome-dependent.

### WRN attenuates CPT-induced cellular senescence

To explore the functional consequences of WRN downregulation in CPT treated cells, we analyzed cell survival and cellular senescence after CPT treatment. Compared to control cells, WRN depleted cells displayed dose-dependent reduction in cell viability in the presence of CPT (Supplementary Figure S4a). However, knockdown of RECQL4 did not increase the sensitivity of cells to CPT. These results suggest a distinct protective role of WRN against the cytotoxic effects of CPT.

**Figure 4:**
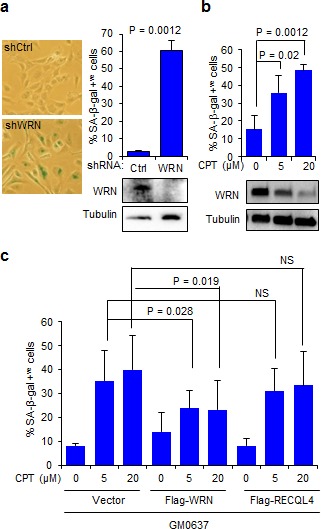
WRN attenuates CPT-induced cellular senescence **a.** Knockdown of WRN induces cellular senescence in human fibroblasts. Left, micrographs showing SA-β-gal stained GM637 cells; right, bar graph showing SA-β-gal positive cells (*n* = three independent experiments). Immunoblots showing knockdown levels of WRN. shCtrl, control shRNA; shWRN, WRN shRNA **b.** CPT-induced cellular senescence in human fibroblasts. Bar graph showing SA-β-gal positive GM637 cells treated with CPT for 18 h. Error bars represent standard deviation from two independent experiments. Immunoblots indicate WRN degradation after CPT treatment. **c.** Ectopic expression of WRN attenuates CPT-induced cellular senescence. GM637 cells transfected with vector, Flag-WRN and Flag-RECQL4 were treated with indicated CPT concentrations and stained for SA-β-gal (*n* = three independent experiments).

It is well established that DNA damage induces cellular senescence [20], and we and others have shown that WRN plays a role in preventing this process [21, 22]. Because the results presented above showed that WRN is downregulated in CPT-treated cells we hypothesized that decreased abundance of WRN could exacerbate CPT-induced senescence. To test this possibility, we measured senescence-associated β-galactosidase (SA-β-gal) activity, a marker of senescence [21], in fibroblasts and U2OS cells treated with CPT. Knockdown of WRN in human fibroblasts, GM0637, induced cellular senescence in ~60% of the cell population (Figure 4a). Treatment of cells with 5 and 20 μM CPT, levels that downregulated WRN, also induced senescence in ~35 and ~45% of cells, respectively (Figure 4b). To evaluate any protective role(s) that WRN may provide against CPT-induced cellular senescence, GM0637 cells were transfected with 3xFlag-WRN (Supplementary Figure S4b) for 24 hours before treatment with CPT. Consistent with the results obtained in the untransfected cells (Figure 4b), vector-transfected cells exposed to 5 and 20 μM CPT accumulated ~35 and ~40% senescent cells. However, vector-transfected cells treated with DMSO only accumulated ~8% senescent cells (Figure 4c) and the ability of CPT to induce the senescent phenotype was attenuated in cultures expressing 3xFlag-WRN (Figure 4c). The 3xFlag-WRN expressing cultures contained ~14% and ~24% senescent population in the presence of DMSO and CPT, respectively (Figure 4c). Similar results were observed in U2OS cells expressing C-terminal GFP-tagged WRN (Supplementary Figure S4c). However, ectopic expression of 3xFlag-RECQL4 (Supplementary Figure S4b) did not significantly inhibit CPT-induced cellular senescence (Figure 4c). These results demonstrate that WRN provides partial protection against CPT-induced cellular senescence.

### WRN degradation and CPT sensitivity in breast cancer cells

A specific mutation (Phe1074Leu) in WRN is found to increase the risk of breast cancer incidence [23, 24], and dysregulation of WRN expression is observed in breast cancer cells lines [5, 6]. Lately there has been an interest in using Top1 inhibitors in the treatment of breast cancer [25, 26], which traditionally has been treated with other approaches. Since Top1 protein expression is tightly correlated with mRNA expression in various cancer cell lines [27] and the above results show that WRN and Top1 are targets for degradation in CPT treated cells, we conducted an *in silico* search to find correlations between the steady-state expression of *Top1* and *WRN* and the sensitivity to Top1 inhibitors using Cancer Cell Line Encyclopedia (CCLE) and Cancer Genome Atlas (TGCA) databases. The results suggest that, irrespective of cancer types, toptecan sensitivity is generally associated with *Top1* (*p*-value = 7.7e-03) and *WRN* (*p*-value = 2.27e-06) mRNA expression (Supplementary Figure S5a, S5b and 5c). Additionally, mining of breast cancer specific RNAseq data from TCGA, indicated a strong correlation between *Top1* and *WRN* expression (Supplementary Figure S5a; r = 0.325, *p* = 5.83e-26) suggesting the importance of these two genes in breast cancer.

**Figure 5:**
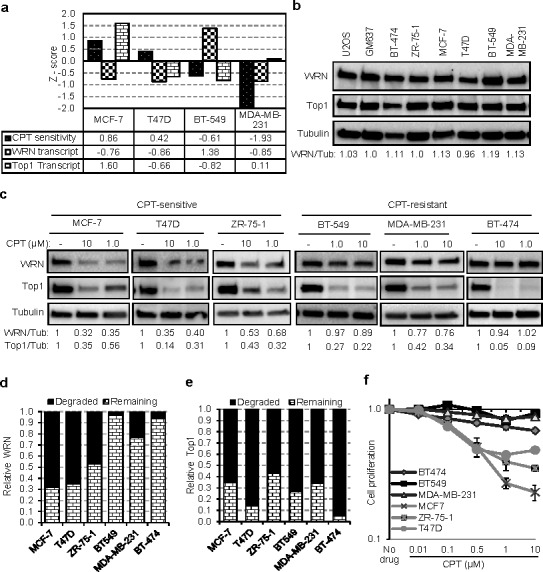
WRN degradation is associated with sensitivity of tumor cells to CPT **a.** WRN expression and CPT sensitivity of breast cancer cells lines. Graph showing Z scores of WRN transcript expression and CPT sensitivity as documented in NCI-60 Cell Miner database. Z score for WRN transcript intensity indicates relative mRNA expression compared to average expression of NCI-60 panel of cell lines. Negative Z score for CPT sensitivity indicates the resistance of the cell line to CPT. **b.** WRN protein expression in tumor cell lines. **c.** CPT-dependent WRN degradation in CPT-sensitive and CPT-resistant breast cancer cell lines. Immunoblots represents protein levels in cells treated with CPT for 16 h. Graphs showing relative degradation of WRN **d.** and Top1 **e.** in breast cancer cell lines treated with 10 μM CPT. **f.** CPT-dependent cell viability and proliferation in sensitive and resistant breast cancer cells. Graph showing relative cell proliferation after 24 h of CPT (0.01 to 10 μM) treatment. Error bars represent SEM from three independent experiments.

To investigate WRN and Top1 expression and the phenomenon of CPT-induced WRN degradation in breast cancer cells, we used three CPT-sensitive (MCF-7, T47D and ZR-75-1) and three CPT-resistant (BT549, MDA-MB-231 and BT-474) cell lines. The Cell Miner database (http://discover.nci.nih.gov/cellminer/) information on the breast cancer cell lines showed that WRN expression was low in two of the CPT-sensitive cell lines (MCF-7 and T47D), high in one CPT-resistant cell line (BT-549) and low in another CPT-resistant (MDA-MB-231) cell line (Figure 5a). Western blot analysis of WRN in the six breast cancer cell lines showed that these cells expressed WRN at relatively similar levels (within 20% of each other: Figure 5b). However, upon treatment with CPT, a significant difference in WRN degradation was observed between the CPT-sensitive and -resistant cell lines (Figure 5c). The CPT-sensitive cells showed significant WRN degradation after CPT treatment, whereas the CPT-resistant cells showed minimal WRN degradation (Figure 5c and 5d). In contrast, CPT-induced Top1 degradation was extensive in both (Figure 5c and 5e) demonstrating that Top1 has no predictive power and further that dose was sufficient to induce degradation in all cell lines.

A seminal study reported a high degree of heterogeneity in Top1 degradation after CPT treatment [28]. Our results confirm this observation in MCF-7, T47D, HCT116, and WI38 cell lines treated with CPT for 8 h (Supplementary Figure S6a); however Top1 was extensively degraded in all tested cell lines, including ZR-75-1, after 16 h of treatment (Figure 5c and Supplementary Figure S6a and S6b). Supporting the importance of WRN in cell proliferation after DNA damage, CPT-sensitive breast cancer cells, which displayed drug-induced WRN degradation, showed compromised cell proliferation after CPT treatment (Figure 5f). Cell survival and proliferation of CPT-resistant cells was largely unaffected by CPT (Figure 5f). Together these results demonstrate that CPT-sensitivity correlated with WRN degradation and not Top1 degradation.

### *Top1* and *WRN* mRNA expression in human breast cancers

**Figure 6:**
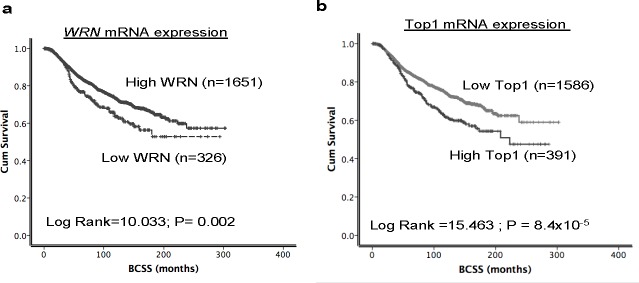
Top1 and WRN mRNA expression in METABRIC cohort Kaplan Meier curves showing BCSS (Breast cancer specific survival) based on Top1 expression **a.** and WRN expression **b.**

Given the essential role of WRN and Top1 in DNA repair and replication we studied their role in breast cancer pathogenesis and prognosis. Inhibition of WRN promoter methylation increased the WRN mRNA levels which in turn increased the protein levels in several cancer cells [6], and a tight correlation between Top1 mRNA and protein expression was observed in the NCI-60 panel of cell lines [27] implying that high mRNA leads to high protein expression. Therefore we investigated the mRNA levels of *WRN* and *Top1* in the METABRIC (Molecular Taxonomy of Breast Cancer International Consortium) cohort. Primary tumors were expression profiled [29] to investigate associations with clinic-pathological parameters and survival in patients. Nearly 16.5% (326/1977) of tumors had low *WRN* mRNA expression and ~85.5% (1651/1977) tumors had high *WRN* mRNA expression (Figure 6a). Low *WRN* mRNA expression was significantly associated with aggressive clinicopathological features including high histological grade, lymph node stage, high risk Nottingham prognostic index (NPI) > 3.4 and Her-2 over expression (ps≤0.01) (Table 1). Interestingly, ER negative and triple negative phenotypes were more common in tumors with high WRN mRNA expression (ps≤0.01). Low *WRN* mRNA expression was significantly associated with molecular phenotypes: PAM50.Her2, PAM50.LumB, Genufu subtype (ER+/Her2-/High proliferation) and Genufu subtype (Her2 positive) breast tumors (ps≤0.01). On the other hand, PAM50.LumA tumors, PAM50.basal and Genufu subtype (ER+/Her2-/low proliferation) express high levels of *WRN* mRNA (ps≤0.01). Similarly, the *WRN* mRNA level was significantly associated with the various integrative clusters (Table 1) described in the METABRIC study which was based on gene copy number changes and gene expression data [29]. Low *WRN* mRNA expression was significantly associated with clusters, which had poor clinical outcome in the METABRIC study [29]. Low *WRN* mRNA expression in tumors was associated with adverse Breast Cancer Specific Survival (BCSS) in the whole cohort (*p* = 0.002) (Figure 6a). The data provides evidence that low WRN mRNA expression is associated with aggressive phenotypes and poor survival in patients.

**Table 1:** Clinico-pathological characteristics of METABRIC cohort based on *WRN* and *Top1* expression

Variable	*WRN* mRNA Expression		P Values	*Topo I* mRNA Expression		P Values
	Low	High		Low	High	
		n (%)		n (%)	n (%)		n (%)
Lymph node stage						
Negative	139 (42.8)	896 (54.4)	0.001	835 (52.8)	200 (51.3)	0.389
Positive (1-3)	65 (20.0)	249 (15.1)		243 (15.4)	71 (18.2)	
Positive (> 3)	121 (37.2)	501 (30.4)		503 (31.8)	119 (30.5)	
Grade						
G1	17 (5.4)	152 (9.6)	0.002	155 (10.2)	14 (3.8)	2.2×10^−10^
G2	113 (36.1)	657 (41.7)		656 (43.1)	114 (31.0)	
G3	183 (58.5)	767 (48.7)		710 (26.5)	240 (64.9)	
Tumour Size (cm)						
T 1a+b (1.0)	14 (4.3)	78 (4.8)	0.073	79 (5.0)	13 (3.3)	0.021
T 1c (> 1.0-2.0)	106 (32.9)	660 (40.8)		634 (40.4)	132 (33.9)	
T2 (> 2.0-5)	183 (56.8)	818 (50.0)		782 (49.9)	219 (56.3)	
T3 (> 5)	19 (5.9)	79 (4.8)		73 (4.7)	25 (6.4)	
NPI						
≤ 3.4	46 (14.8)	342 (21.9)	0.005	345 (22.9)	47 (12.8)	2.0×10^−5^
> 3.4	265 (85.2)	1217 (78.1)		1162 (77.1)	320 (87.2)	
Her2 over expression						
No	264 (81.0)	1468 (88.9)	7.1×10^−5^	1424 (89.8)	308 (78.8)	3.2×10^−9^
Yes	62 (19.0)	183 (11.1)		162 (10.2)	83 (21.2)	
ER						
Negative	60 (18.4)	410 (24.8)	0.013	354 (22.3)	116 (29.7)	0.002
Positive	266 (81.6)	1241 (75.2)		1232 (77.7)	275 (70.3)	
PgR						
Negative	155 (47.5)	781 (47.3)	0.936	716 (45.1)	220 (56.3)	8.0×10^−5^
Positive	171 (52.5)	870 (52.7)		870 (54.9)	171 (43.7)	
*Genefu subtype*						
ER-/Her2 -	9 (5.5)	141 (17.0)	1.7×10^−4^	124 (15.5)	26 (13.1)	0.538
ER+/Her2-/high proliferation	81 (49.4)	285 (34.3)	2.6×10^−4^	279 (76.2)	87 (23.8)	0.053
ER+/Her2-/low proliferation	45 (27.4)	323 (38.9)	0.005	326 (40.9)	42 (21.2)	1.0×10^−5^
Her-2 positive	29 (17.7)	81 (9.8)	0.003	67 (8.4)	43 (21.7)	4.9×10^−7^
*PAM50 subtype*						
PAM50.Her2	52 (17.9)	186 (12.6)	0.014	159 (11.3)	79 (21.4)	4.4×10^−7^
PAM50.Basal	28 (9.7)	302 (20.4)	1.8×10^−5^	266 (19.0)	64 (17.4)	0.478
PAM50.LumA	98 (33.8)	617 (41.6)	0.013	613 (43.7)	102 (27.6)	2.0×10^−6^
PAM50.LumB	112 (38.6)	377 (25.4)	4.0×10^−6^	365 (26.0)	124 (33.6)	0.004
*IntClust subgroups*						
intClust.1	35 (10.7)	102 (6.2)	0.003	95 (6.0)	42 (10.7)	0.001
intClust.2	17 (5.2)	55 (3.3)	0.097	54 (3.4)	18 (4.6)	0.257
intClust.3	28 (8.6)	262 (15.9)	0.001	251 (15.8)	39 (10.0)	0.003
intClust.4	32 (9.8)	311 (18.8)	8.5×10^−5^	308 (19.4)	35 (9.0)	9.7×10^−7^
intClust.5	53 (16.3)	136 (8.2)	7.0×10^−6^	118 (7.4)	71 (18.2)	1.1×10^−10^
intClust.6	29 (8.9)	57 (3.5)	1.1×10^−5^	66 (4.2)	20 (5.1)	0.408
intClust.7	42 (12.9)	147 (8.9)	0.026	157 (9.9)	32 (8.2)	0.302
intClust.8	30 (9.2)	270 (16.4)	0.001	251 (15.8)	49 (12.5)	0.104
intClust.9	48 (14.7)	98 (5.9)	2.9×10^−8^	117 (7.4)	29 (19.9)	0.978
intClust.10	12 (3.7)	213 (12.9)	2.0×10^−6^	169 (10.7)	56 (14.3)	0.041

HER2: human epidermal growth factor receptor 2; ER: estrogen receptor; PgR: progesterone receptor; Triple negative: ER−/PgR−/HER2−.

In the METABRIC cohort, ~20% (390/1977) of tumors had high *Top1* mRNA expression and 80% (1586/1977) tumors had low *Top1* mRNA expression (Figure 6b). High *Top1* mRNA expression was significantly associated with aggressive clinicopathological features including high histological grade, larger tumor size, high risk Nottingham prognostic index (NPI) > 3.4, Her2 over expression, and ER- and PR- tumors (ps < 0.05) (Table 1). High *Top1* mRNA expression in tumors was associated with adverse BCSS in the whole cohort (*p* = 0.002) (Figure 6b). Of note, patients in the METABRIC cohort did not receive Top1 inhibitor based therapy. Nevertheless, taken together, the data provides evidence that high *Top1* mRNA expression is associated with aggressive breast cancers.

### WRN and Top1 co-expression in ER positive and ER negative breast cancers

Our *in vitro* results suggest that WRN degradation could be a useful marker in personalized chemotherapy. To identify potential patient populations that may benefit from CPT-based regimens, we proceeded to sub-group analysis of patients in the METABRIC cohort where ER positive tumors comprised of 1507/1977 (76.2%) of tumors and 470/1977 (23.8%) were ER negative tumors.

**Figure 7:**
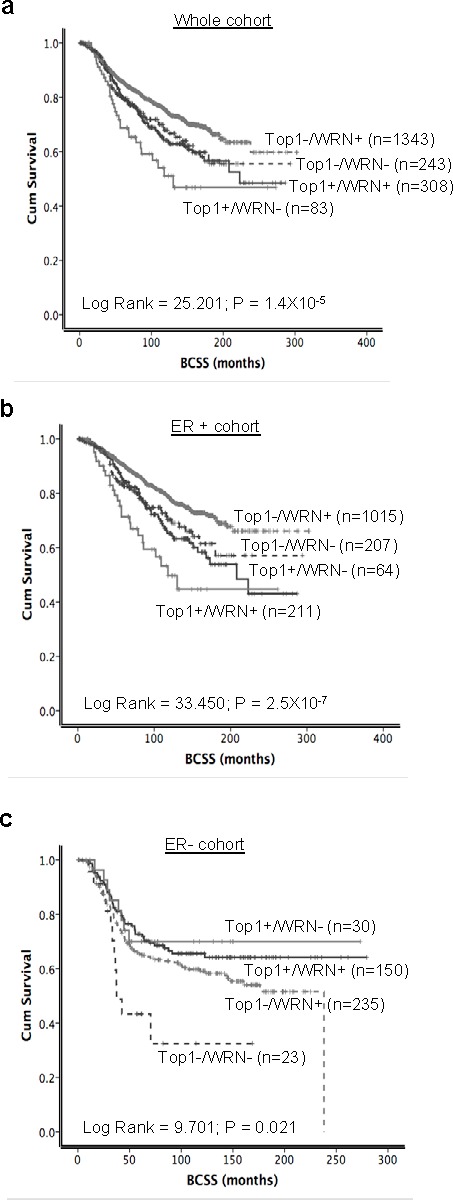
Kaplan Meier curves showing BCSS with WRN and Top1 expression **a.** WRN and Top1 expression in whole cohort. **b.** and **c.** combined expression of WRN and Top1 in ER positive and ER negative METABRIC cohorts.

When Top1 and WRN were combined together, as expected, patients with high WRN/high Top1 expressing tumors had worst survival compared to tumors with low WRN/low Top1 expression in the whole cohort (*p* < 0.0001) (Figure 7a and Supplementary Table 1) as well as in the ER positive cohort (*p* < 0.0001) (Figure 7b). In addition, in high risk ER positive tumors that received adjuvant endocrine therapy, high WRN/high Top1 expressing tumors was associated with poor survival (*p* < 0.001) (Supplementary Figure S7a). In ER positive tumors that received no endocrine therapy, high WRN/high Top1 expressing tumors remains associated with poor survival compared to tumors with low WRN/low Top1 expressing (*p* = 0.006) (Supplementary Figure S7b).

Interestingly, when WRN and Top1 examined were combined in ER negative tumors, low WRN/low Top1 expressing tumors had the worst survival compared to patients with high WRN/high Top1 expressing tumors (*p* = 0.021) (Figure 7c). Collectively, the data suggest that WRN/Top1 expression may have predictive significance in the ER negative sub-group. In patients who had no adjuvant chemotherapy, compared to tumors with low WRN/low Top1 expression, tumors with high WRN/high Top1 expression have a poorer prognosis (*p* = 0.043) (Supplementary Figure S7d).

Taken together, in ER positive tumors WRN/Top1 co-expression may have predictive significance in patients who received endocrine therapy and prognostic significance in patients who received no endocrine therapy. Importantly, the data also suggest that an alternative form of therapy such as those targeting Top1 could be a promising strategy in high Top1/high WRN expressing tumors. Overall these results identify that ~14% of ER positive and ~34% of ER negative breast cancer patients express high WRN and high Top1 in their tumors and may benefit from CPT-based personalized chemotherapy.

## DISCUSSION

Cellular studies show that RecQ helicases protect against the cytotoxic effects of CPT [9-13]. To characterize the mechanism, we analyzed the expression and stability of the human RecQ helicases in cells exposed to CPT. CPT affects the degradation of WRN protein without altering expression of WRN mRNA or the abundance of the other RecQ proteins. CPT treatment resulted in re-localization of WRN from the nucleolus to the nucleoplasm and cytoplasmic organelles, and resulted in downregulation of the protein in a time- and CPT concentration-dependent manner. CPT-induced WRN downregulation was mediated by the ubiquitin proteasome pathway, and ectopic expression of WRN attenuated the CPT-induced senescent phenotype. Drug-induced WRN degradation in breast cancer cell lines was associated with the sensitivity of cells to CPT. Breast cancer patients with ER-positive tumors expressing high WRN and Top1 had poor survival. Overall findings presented in this study, based on cellular studies, *in silico* studies and clinical studies, suggest that WRN helicase along with Top1 can be targeted with CPT at the protein level and could be a used as a potential marker for predicting the efficiency of CPT-based chemotherapy for breast cancer.

WRN, the largest human RecQ protein, contains a nuclease domain, which is not found in other RecQ proteins, and catalyzes four DNA-dependent reactions: 3′-5′-exonuclease, ATPase, DNA strand annealing and 3′-5′-helicase activities. Through its enzymatic functions, WRN acts on various DNA structures to facilitate DNA repair. Mutations in *WRN* leads to defects in DNA repair, premature aging and to cancer susceptibility [23, 30, 31]. Small molecules targeting DNA repair proteins have profound effects in inhibiting tumor survival. A recent study identified NSC 19630 [1-(propoxymethyl)-maleimide] as a specific inhibitor of WRN, which synergistically inhibited cell proliferation and induced DNA damage with topotecan [32].

CPT and its analogs are used against a broad spectrum of tumors [33]. It covalently binds to the interface between Top1 and DNA, thereby blocking the cleavage/re-ligation activities of the topoisomerase [7]. As a result high loads of protein-linked DNA breaks block replication and transcription events and initiate senescence and cell death [8, 34]. The novel observation reported here is that WRN is specifically degraded in CPT-treated cells exacerbating the adverse consequences of CPT-induced DNA damage and loss of functional Top1. CPT treatment leads to a reduction of Top1 protein, trapped as the CPT-DNA-Top1 complex, by small ubiquitin-like modifier (SUMO) protein conjugation and by the ubiquitin proteasome pathway [18, 35, 36]. As shown here, CPT also causes the specific degradation of WRN, but not of the other RecQ helicases, likely because WRN physically and functionally interacts with Top1 [17]. Thus, the physical association between WRN and Top1 could lead to a shared ‘fate’ of degradation in CPT-treated cells. However, as shown in Figure 5c, Top1 degradation can be uncoupled from WRN degradation in certain cell types.

Protein degradation is orchestrated mainly by the proteasome and lysosome proteolytic systems. Lack of KFERQ motifs in WRN suggests that it may not be targeted for degradation by lysosomes. Our results showed that CPT treatment induced re-localization of WRN from the nucleolus to the cytoplasm where ubiquitin-dependent protein turnover occurs. Ubiquitinylation of proteins occur by the addition of ubiquitin molecules to lysine residues on target proteins. In the G1 phase of the cell cycle, BLM is ubiquitinated and degraded to promote non-homologous end joining [37]. Such degradation pathways might be operating on the other RecQ helicases depending on the cell cycle, as well as the functional contexts. Acetyltransferases CBP and p300 increased the stability of WRN in the presence of mitomycin C, and WRN stability and degradation was respectively associated with acetylation and ubiquitination of six lysine residues of the protein [15]. We observed ubiquitination of WRN after CPT treatment, suggesting decreased WRN stability due to protein degradation by the ubiquitin-proteasome pathway. However, the ubiquitin ligases that mediate CPT-dependent ubiquitination of WRN are yet to be identified. Interestingly, CPT-induced WRN degradation was confined to CPT-sensitive cancer cells suggesting a role for WRN in rendering resistance against CPT. In addition to enhancing the enzymatic activity of Top1 [17], WRN's nuclease and helicase activities might play critical roles in removing CPT-Top1-DNA cytotoxic lesions to reduce cytotoxic effects of the drug. Therefore it is possible that the WRN contributes to resistance to CPT by enhancing DNA repair capabilities at various levels.

Senescence is enhanced when RecQ helicases are deficient [21], or if there is persistent DNA damage [38] and after treatment with anticancer drugs like CPT [39]. In our recent report we demonstrated that knockdown of RecQ helicases (with the exception of RECQL1) resulted in the accumulation of DNA-SCARS (DNA segments with chromatin alternations reinforcing senescence), increased p21 and p16 along with increased SA-β-gal activity [21]. WRN knockdown and CPT treatment both induce DNA damage and cause increased p21 expression and SA-β-gal activity [39]. Here, we demonstrate that WRN degradation and increased senescence after CPT treatment can be suppressed by ectopically expressing WRN. Rescue of WRN in CPT-treated cells might increase the efficiency of DDR to remove cytotoxic DNA lesions.

Top1 gene amplification has been reported in about 30% of breast cancers [40] and a tight correlation between mRNA and protein expression was observed in the NCI-60 panel of cell lines [27]. Irinotecan, a semisynthetic derivative of CPT is routinely used in colorectal cancer therapy [41]. Although phase II trials have demonstrated a response rate of about 5% to 23%, irinitotecan is not routinely used as monotherapy in breast cancer [26]. However, etirinotecan, a long acting derivative of irinotecan has shown a response rate of 29% and is currently being evaluated in a large phase III clinical trial in metastatic breast cancer [25]. Given the potential promise of Top1 inhibitors in breast cancer, the development of predictive biomarkers to personalize therapy is highly desirable. In the current study, we suggest that CPT-induced WRN degradation in breast cancer cells could be a biomarker for CPT sensitivity. Taken together, the pre-clinical data show that *WRN* and/or *Top1* expression could have prognostic and/or predictive significance in breast cancers. To explore this hypothesis, we conducted the first large study of *WRN* and *Top1* expression in human breast cancers. We observed that *Top1* mRNA overexpression was associated with aggressive highly proliferative breast cancers. In contrast, for *WRN*, low mRNA expression was associated with adverse clinicopathological features and was linked to poor breast cancer specific survival. Taken together, these novel observations suggest that low *WRN* expression in human tumors may promote a ‘mutator phenotype’ leading to aggressive breast cancers. Although the mechanism of regulation of *WRN* mRNA expression is not understood, Agrelo et al. have previously shown that epigenetic inactivation of WRN is frequent in solid tumors with the highest prevalence in colorectal tumors [37.9% (69/182 tumors)] [6]. In a small cohort of breast tumors (*n* = 58), 17.2% (10/58) showed WRN inactivation although the authors did not describe any clinicopathological associations in that study [6]. In our study, we have found that *WRN* mRNA expression level was low in 326/1977 of breast tumors (16.5%) which concur strikingly with the study by Agrelo et al. In colorectal cancers, WRN hypermethylation and depletion is associated with good response to irinotecan therapy [6]. We speculate that low WRN expression could result in a genomic instability phenotype with an aggressive behavior. To support this hypothesis, we observed that low WRN was associated with lymph node positivity, grade 3 and HER-2 overexpression. As the clinical study presented here provides prognostic information, future studies, particularly in patients who receive neoadjuvant chemotherapy (including Top1 inhibitor) could provide predictive information.

When *WRN* and *Top1* were combined, surprisingly we found that tumors with high *Top1*/high *WRN* expression have poor survival, particularly in the ER positive sub-group. Top1 has essential roles during replication and proliferation. Highly proliferative ER positive breast tumors (PAM50. Lum B phenotype) manifest endocrine resistance. As high Top1 expression is significantly associated with PAM50. Lum B breast tumors (Table 1), we speculate that endocrine resistance may be contributing to the poor survival seen in patients. The data for WRN is intriguing. Whether WRN-mediated DNA repair in ER positive tumors would influence therapy outcome remains to be established. Interestingly, high expression of the multifunctional WRN helicase is observed in several cancer cell lines (Supplementary Figure S5 and [5]). As WRN participates in various DNA repair pathways, it is possible that it enhances the DNA repair capabilities of established tumor cells to withstand DNA damage induced by endogenous and exogenous agents.

In conclusion, we provide the first pre-clinical evidence that WRN degradation is a biomarker of CPT sensitivity in breast cancer cells, where it can distinguish CPT- sensitive and resistant breast cancer cells. Therefore, WRN expression and degradation could be used to identify tumors which may be sensitive to CPT and its derivatives. However, a prospective clinical trial of Top1 inhibitor therapy in ER positive breast cancers would be required to confirm our *in vitro* results. Additionally we show that in human breast cancers, *Top1* and/or *WRN* expression has prognostic and predictive significance. *Top1*/*WRN* expression based stratification could be a promising approach to personalize Top1 inhibitor therapy in breast cancer patients.

## MATERIALS AND METHODS

### Cell culture

U2OS, 293T, GM637, BT-474, BT-549, MCF-7, MDA-MB-231, T47D, ZR-75-1, HCT116, HCT116 p53^−/−^ and WI-38 were cultured as described [14, 21, 28]. For knockdown experiments, cells were infected with lentivirus carrying either control or WRN or RECQL4 small hairpin RNA (shRNA) as described before [21] for at least 72 hours. For overexpression, transfections were performed with 3 × 10^5^ cells using 1 μg of pCMV-tag4 plasmids with 3xFlag or 3xFlag-WRN or 3xFlag-RECQL4, or pEGFP, pEGFP-WRN, or pcDNA3.1-HA-ubiquitin using jetPRIME (Polyplus transfection) [21]. CPT (Sigma) was dissolved in DMSO, and 10 mM aliquots were stored at −20°C, and cells were treated at 10 μM unless otherwise specified. For proteasome inhibition, cells were treated with 10 μM MG132 (EMD Millipore) and for inhibiting protein synthesis, cells were pretreated with 10 μg/ml cycloheximide (CHX; Sigma). For detecting lysosomes, cells were infected with CellLight Lysosome-GFP baculovirus (Life technologies). For IR treatments, cells were grown in 6 cm plates and irradiated with 1 or 10 gy of ^137^Cs gamma rays.

### Immunoblotting

Cells were lysed in either IP lysis buffer [14] or RIPA buffer (Thermo Scientific) and briefly sonicated. The Subcellular Protein Fractionation kit for Cultured Cells (Thermo Scientific) was used to isolate proteins from specific cellular compartments i.e. cytoplasm, cytoplasmic organelles, nuclear soluble and chromatin. Proteins were detected by Western blotting [14] using in house (anti-WRN, -RECQL4, -RECQL5), Abcam (anti-CtIP, -XLF), Millipore (histone 3), BD Pharmingen (anti-53BP1, -calretuculin, -Top1) and Santa Cruz (anti-RECQL1, -Ku80, -actin, -tubulin) antibodies and quantitated by using ImageJ 1.46r software (National Institutes of Health, USA).

### Quantitative real-time polymerase chain reaction (qRT-PCR)

Total cellular RNA was isolated using RNeasy RNA isolation kit (Qiagen) from 500,000 cells treated with or without CPT. Complementary DNA (cDNA) synthesis and qRT-PCR were performed in a single reaction tube with 15 ng of RNA using gene-specific primers and SuperScript III Platinum SYBR Green One-Step qRT-PCR kit (Life Technologies). Reverse transcription and qRT-PCR were performed according to manufacturer's protocol in a 7900 Fast Real-Time PCR system (Applied Biosystems). Hs00262956_m1 (RECQL1), Hs00172060-m1 (BLM), Hs01087915_m1 (WRN), Hs01548660_g1 (RECQL4), Hs00188633_m1 (RECQL5) and Hs02758991_g1 (GAPDH) primer sets (Applied Biosystems) were used for quantitating mRNA.

### Immunostaining and microscopy

Cells grown in chambered glass slides (Labtek) for 24 hours in the presence or absence of bacullovirus expressing lysosome specific GFP were treated with CPT. Post treatment, cells were fixed and immunostained for WRN and 53BP1 as described before [21] and imaged with a Zeiss LSM510 (40X objective) microscope and Volocity 3D image analysis software (Perkinelmer). Time-lapse microscopy was performed with U2OS cells stably expressing YFP-WRN using the above mentioned microscope settings supported with temperature and CO_2_-regulated incubation chamber.

### Cell survival and proliferation assays

Cells (5000/well) were seeded in triplicates in a 96-well plate and treated with CPT for 24h. Cell viability and proliferation were assayed according to the manufacturer's protocol using WST-1 reagent (Roche). Cell viability was calculated from three independent experiments and normalized to no treatment controls.

### SA-β-gal assay

Three biological repeats of SA-β-gal staining was performed with control, WRN and RECQL4 expressing cells treated with CPT for 18 hours as previously described [21].

### *WRN* and *Top1* mRNA expression in patient tumors

The METABRIC study protocol, molecular profiling, Tumor Marker Prognostic Studies (REMARK) criteria were followed [29, 42] to study *WRN* and *Top1* mRNA expression. Gene copy number was assayed on the Affymetrix SNP 6.0 platform (data available through the European Genotype Archive, http://www.ebi.ac.uk/ega/page.php under accession Number: EGAS00000000082). The mRNA expression was analyzed as before [29, 43] by using WRN (ILMN_1679881) and Top1 (ILMN_2192316) specific probes.

Breast cancer specific survival (BCSS) was defined as the number of months from diagnosis to the occurrence of BC related-death and the data was analyzed using SPSS (SPSS, version 17 Chicago, IL) as described before [43]. Cumulative survival probabilities were estimated using the Kaplan-Meier method, and differences between survival rates were tested for significance using the log-rank test and multivariate analysis for survival was performed using the Cox proportional hazard model as described before [43].

## SUPPLEMENTARY MATERIAL FIGURES AND TABLE


