# Supplementary material for: Camptothecin targets WRN protein: mechanism and relevance in clinical breast cancer
Source: Oncotarget. 2016 Mar 3;7(12):13269–84. doi: 10.18632/oncotarget.7906 (PMC4924640; doi:10.18632/oncotarget.7906)
Supplement: Supplementary file 1 [file oncotarget-07-13269-s001.pdf]

Camptothecin targets WRN protein: mechanism and relevance in clinical breast cancer

Supplementary Material

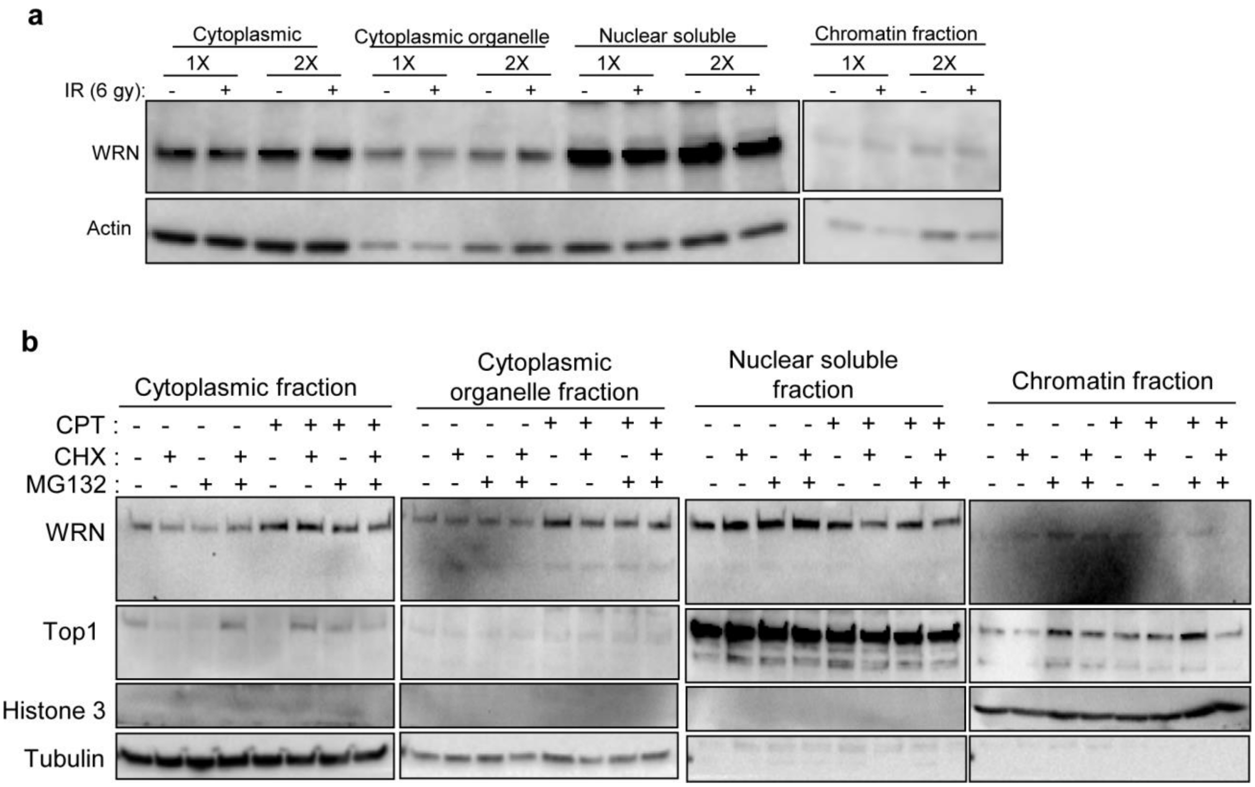

Supplementary figure S1. (a) IR-dependent WRN distribution in the sub-cellular compartments. Western blots showing WRN distribution in sub-cellular compartment of U2OS cells after 3 hour recovery of cells treated with 6 gy of IR. X = loading volume (¼ of lysis buffer used). (b) CPT-induced re-localization of WRN is not significantly affected by pretreatment of cells with cyclohexamide and MG132. Immunoblots showing WRN in the cytoplasmic, cytoplasmic organelle, nuclear soluble and chromatin bound fractions from the cells pretreated with cyclohexamide (10 µg/ml) and MG132 (10 µM) for 1hour and then with 20 µM for 3 hours. Representative blots from three independent experiments.

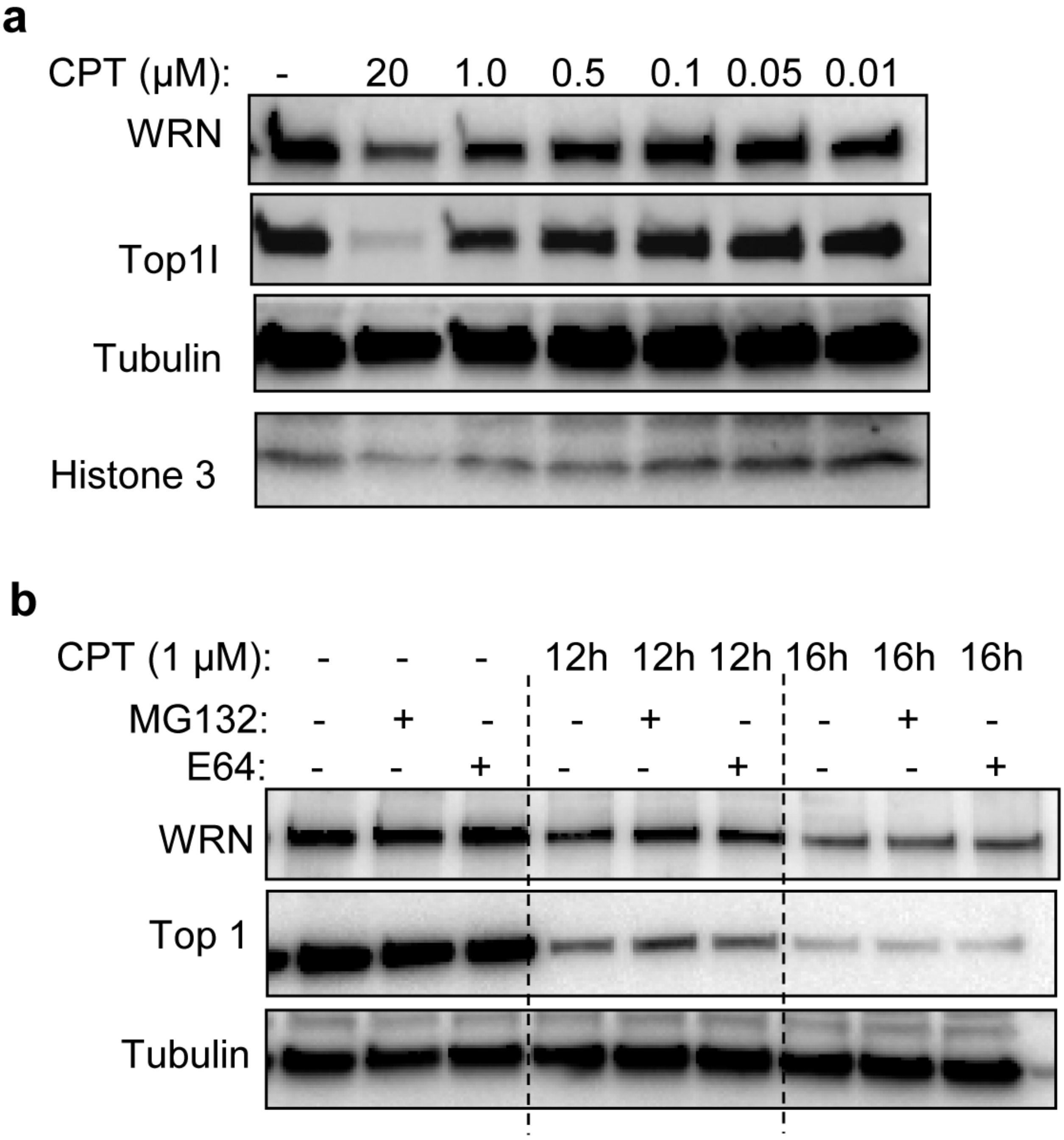

Supplementary figure S2. (a) WRN and Top1 protein expression in cells treated with indicated concentrations of CPT. (b) Inhibition of WRN and Top1 degradation in cells in the presence of proteasome inhibitor MG132 (10 μM).

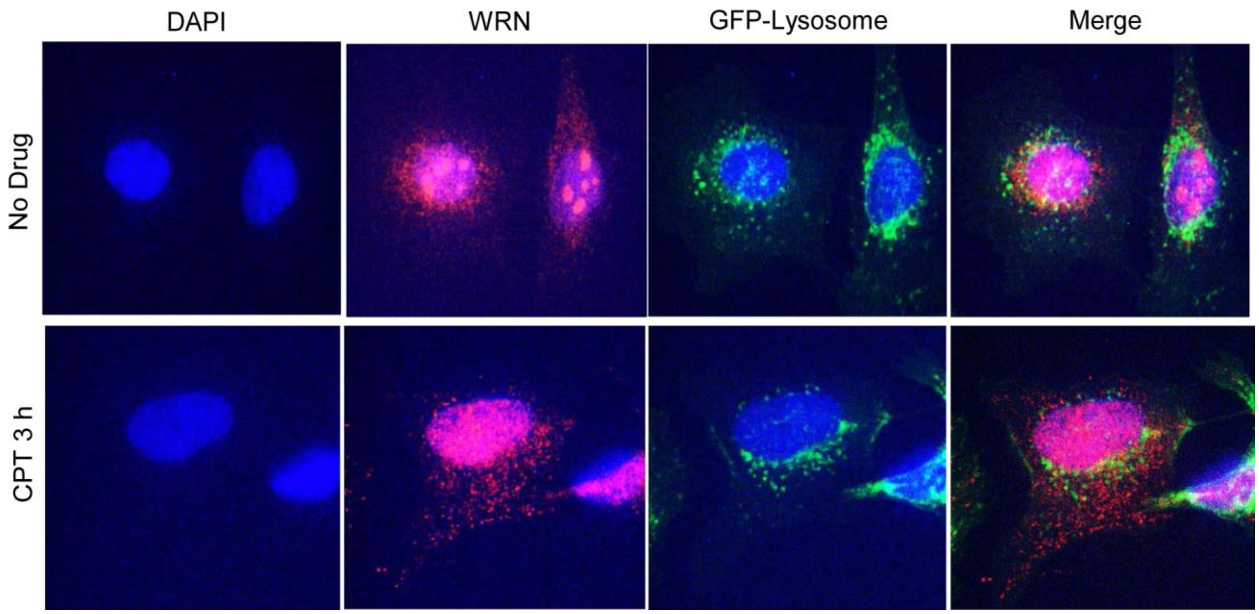

Supplementary figure S3. Localization of WRN to lysosomes in response to CPT and IR. Confocal micrographs of U2OS cells infected with baculovirus expressing lysosome specific GFP for 15 hours followed by CPT (20 μM) treatment for 3 hours and immunostained for WRN.

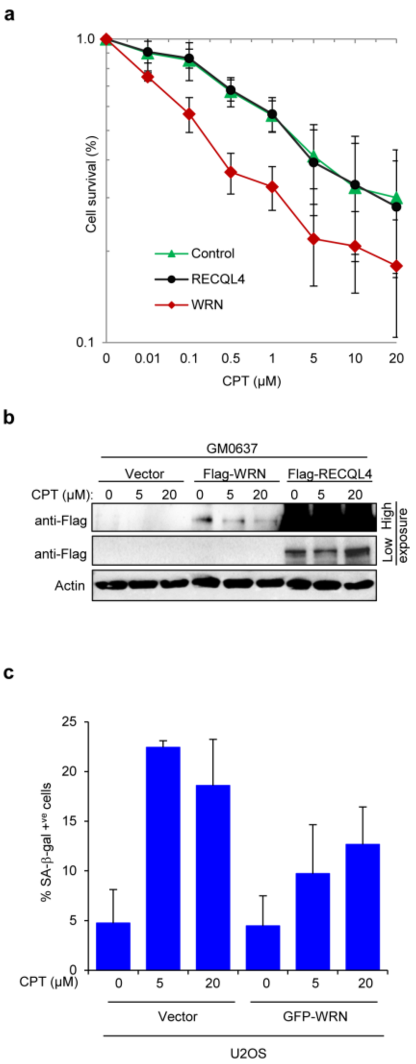

Supplementary figure S4. (a) Knockdown of WRN sensitizes cells to CPT. Graphs represent survival efficiency of WRN and RECQL4 knockdown U2OS cells at 72 h after CPT treatment as measured by cleavage of WST-1. Survival efficiency of CPT treated cells was normalized to untreated cells. Data represents the mean values of four independent experiments and the error bars represent standard deviation. (b) Western blots showing the expression of 3xFlag-WRN and 3xFlag-RECQL4 in GM0637 cells. (c) SA-β-gal positive cells in CPT-treated U2OS cells expressing GFP-WRN. SA-β-gal stained cells quantified as above. (n = two independent experiments).

a

	CCLE correlation with topotecan			TCGA correlation with Top1		
	r	p-value	Bonferoni significance	r	p-value	**Bonferoni significance**
**RECQL1**	**0.14**	**0.00132**	**Yes**	**0.378**	**3.84e-35**	**Yes**
**BLM**	**0.36**	**5.29e-17**	**Yes**	**0.318**	**8.94e-25**	**Yes**
**WRN**	**0.23**	**2.27e-07**	**Yes**	**0.325**	**5.83e-26**	**Yes**
**RECQL4**	**0.21**	**2.31e-06**	**Yes**	**-0.05**	**0.113**	**No**
**RECQL5**	**0.09**	**0.0439**	**No**	**-0.25**	**7.65e-12**	**Yes**
**Top1**	**0.11**	**0.0079**		**1**		

b

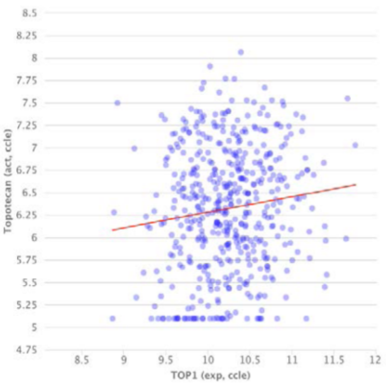

c

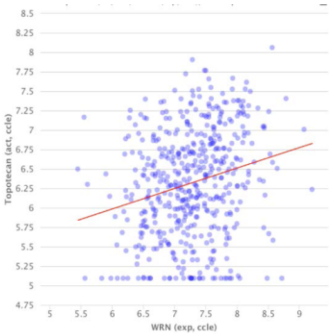

d

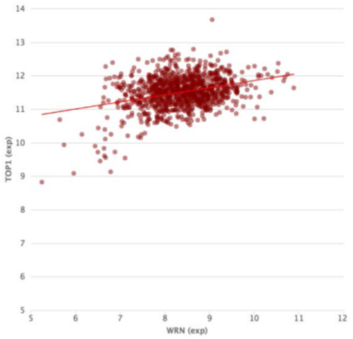

Supplementary figure S5. Bioinformatic analysis of WRN and Top1 expression. (a) *Recql* and *Top1* expression analysis from Cancer Cell Line Encyclopedia (CCLE) and TCGA data base (b) Top1 expression and topotecan sensitivity of cancer cell lines from CCLE database. (c) WRN expression and topotecan sensitivity of cancer cell lines from CCLE database. (d) RNAseq data from breast cancer cells showing association between WRN and Top1.

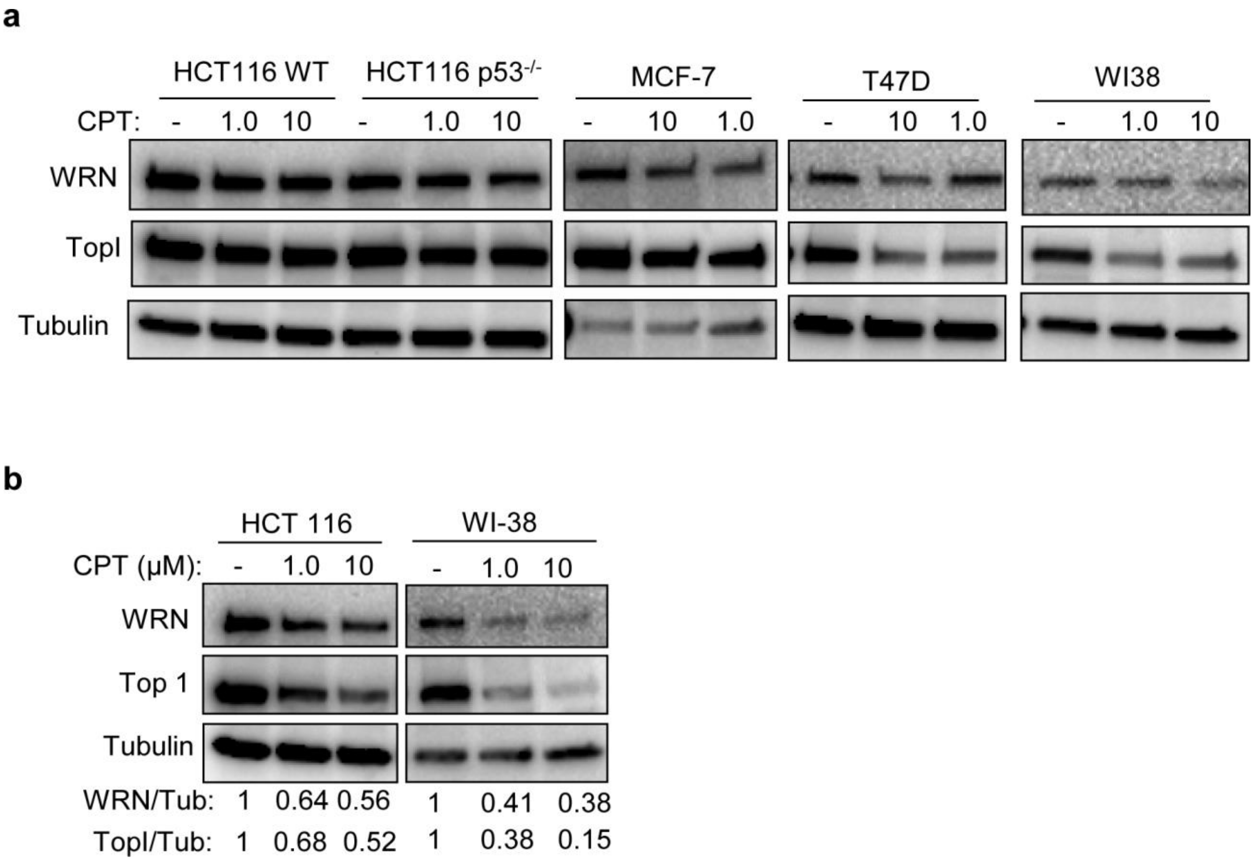

Supplementary figure S6. (a) WRN and Top1 protein levels in indicated cell lines after 8 hours of CPT treatment. (b) WRN and Top1 degradation in CPT-treated HCT 116 and WI-38 cell lines at 16 h.

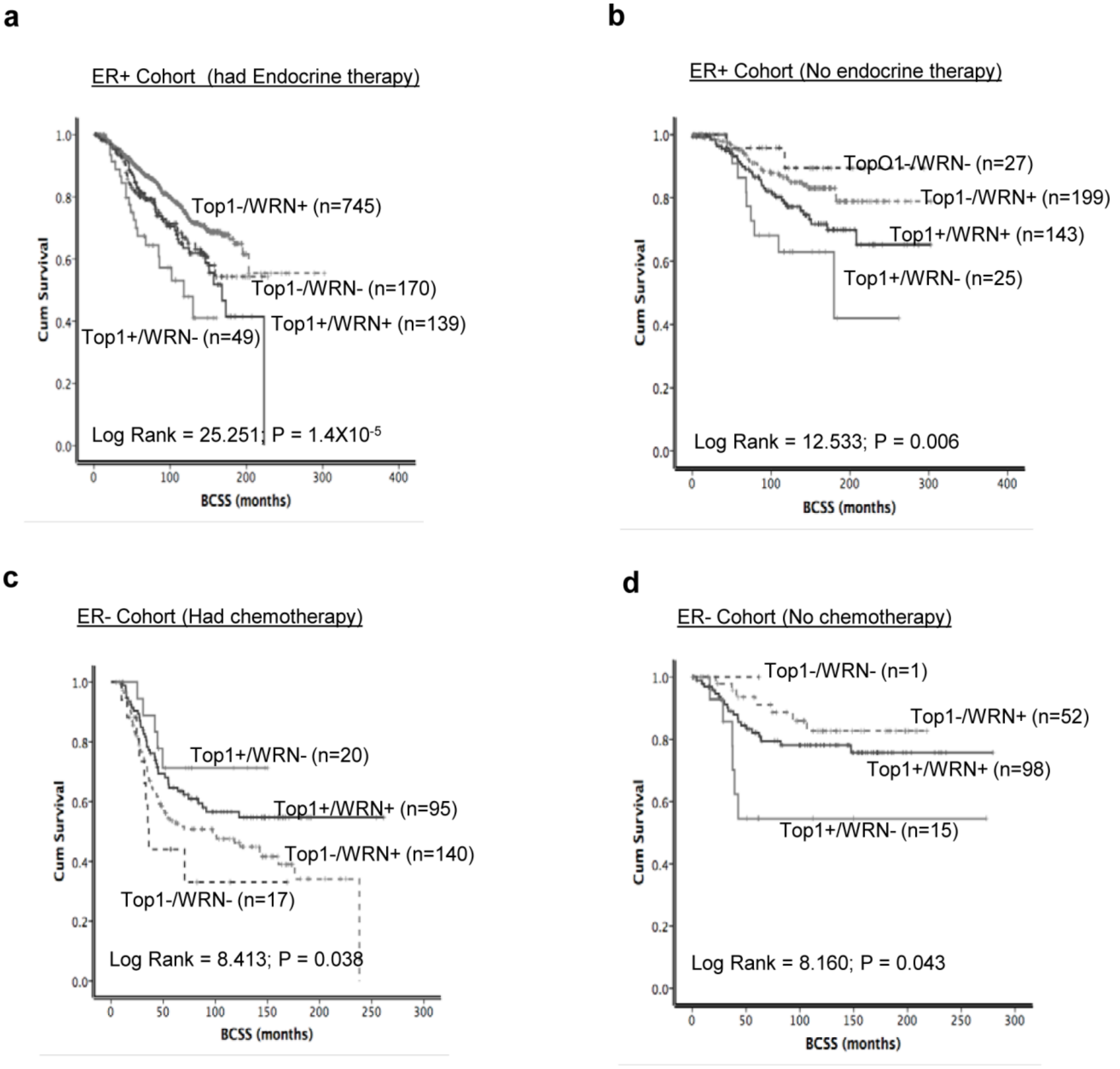

Supplementary figure S7. Kaplan Meier curves showing co-expression of Top1 and WRN. (a) Endocrine therapy received ER positive cohort. (b) ER positive cohort who did not receive endocrine therapy. (c). ER negative cohort who received chemotherapy (d) ER negative cohort with no chemotherapy.

Supplementary Table 1: Association between *TOPO1/WRN* mRNA co-expression and clinico-pathologic variables in METABRIC cohort.

Variable	TOPO1/WRN mRNA Co-expression				P Values
	**TOP1+/WRN+**	**TOP1+/WRN-**	**TOP1-/WRN+**	**TOP1-/WRN+**	
	**N (%)**	**N (%)**	**N (%)**	**N (%)**	
**Pathological Parameters**					
**Lymph node stage**					
Negative	162 (52.8%)	38 (45.8%)	734 (54.8%)	101 (41.7%)	**0.006**
Positive (1-3)	52 (16.9%)	19 (22.9%)	197 (14.7%)	46 (19.0%)	
Positive (>3)	93 (30.3%)	26 (31.3%)	408 (30.5%)	95 (39.3%)	
**Grade**					
G1	10 (3.4%)	14 (3.8%)	155 (10.2%)	14 (3.8%)	**$1.6X10^{-10}$**
G2	94 (32.3%)	20 (26.0%)	563 (43.8%)	93 (39.4%)	
G3	187 (64.3%)	53 (68.8%)	580 (45.1%)	130 (55.1%)	
**Tumour Size (cm)**					
T 1a+b(1.0)	11 (3.6%)	2 (2.5%)	67 (5.0%)	12 (5.0%)	0.065
T 1c(>1.0-2.0)	108 (35.1%)	24 (29.6%)	552 (41.6%)	82 (34.0%)	
T2 (>2.0-5)	170 (55.2%)	49 (60.5%)	648 (48.8%)	134 (55.6%)	
T3 (>5)	19 (6.2%)	6 (7.4%)	60 (4.5%)	13 (5.4%)	
**NPI**					
≤ 3.4	40 (13.8%)	7 (9.1%)	302 (23.8%)	39 (16.7%)	**$2.0X10^{-5}$**
>3.4	250 (86.2%)	70 (90.9%)	967 (76.2%)	195 (83.3%)	
**Her2 overexpression**					
No	249 (80.8%)	59 (71.1%)	1219 (90.8%)	205 (84.4%)	**$1.6X10^{-10}$**
Yes	59 (19.2%)	24 (28.9%)	124 (9.2%)	38 (15.6%)	
**ER**					
Negative	96 (31.2%)	20 (24.1%)	31 (23.4%)	40 (16.5%)	**0.001**
Positive	212 (68.8%)	63 (75.9%)	1029 (76.6%)	203 (83.5%)	
**PgR**					
Negative	175 (56.8%)	45 (54.2%)	606 (45.1%)	110 (45.3%)	**0.001**
Positive	133 (43.2%)	38 (45.8%)	737 (54.9%)	133 (52.7%)	
**Triple Negative**					
Non Triple Negative	252 (81.8)	79 (95.2)	1105 (82.3)	24 (92.2)	**$2.7x10^{-5}$**
Triple Negative	56 (18.2)	4 (4.8)	238 (17.7)	19 (7.8)	
Genefu subtype					
ER-/Her2 -	24 (15.2%)	2 (5.0%)	117 (17.4%)	7 (5.6%)	**0.002**
ER+/Her2-/high proliferation	65 (41.1%)	22 (55.0%)	220 (32.7%)	59 (47.6%)	**$4.5x10^{-4}$**
ER+/Her2-/low proliferation	37 (23.4%)	5 (12.5%)	286 (42.6%)	40 (32.3%)	**$3.3X10^{-7}$**
Her2 positive	32 (20.3%)	11 (27.5%)	49 (7.3%)	18 (14.5%)	**$8.8X10^{-8}$**
PAM50 subtype					
PAM50.Her2	60 (20.5%)	19 (24.7%)	126 (10.6%)	33 (15.5%)	**$1.0X10^{-6}$**
PAM50.Basal	58 (19.9%)	6 (7.8%)	244 (20.5%)	22 (10.3%)	**$3.1x10^{-4}$**
PAM50.LumA	82 (28.1%)	20 (26.0%)	535 (45.0%)	78 (36.6%)	**$5.6X10^{-8}$**
PAM50.LumB	92 (31.5%)	32 (41.6%)	285 (23.9%)	80 (37.6%)	**$3.0x10^{-6}$**
IntClust subgroups					
intClust.1	30 (9.7%)	12 (14.5%)	72 (5.4%)	23 (9.5%)	**$3.2x10^{-4}$**
intClust.2	15 (4.9%)	3 (3.6%)	40 (3.0%)	14 (5.8%)	0.106
intClust.3	32 (10.4%)	7 (8.4%)	230 (17.1%)	21 (8.6%)	**$1.3x10^{-4}$**
intClust.4	32 (10.4%)	3 (3.6%)	279 (20.8%)	29 (11.9%)	**$4.0X10^{-8}$**
intClust.5	49 (15.9%)	22 (26.5%)	87 (6.5%)	31 (12.8%)	**$7.3X10^{-13}$**
intClust.6	12 (3.9%)	8 (9.6%)	45 (3.4%)	21 (8.6%)	**$1.2x10^{-4}$**
intClust.7	25 (8.1%)	7 (8.4%)	122 (9.1%)	35 (14.4%)	**0.050**
intClust.8	43 (14.0)	6 (7.2%)	227 (16.9)	24 (9.9%)	**0.005**
intClust.9	18 (5.8%)	11 (13.3%)	80 (6.0%)	37 (15.2%)	**$8.1x10^{-7}$**
intClust.10	52 (16.9%)	4 (4.8%)	161 (12.0%)	8 (3.3%)	**$2.0x10^{-6}$**

Bold = Statistically significant; HER2: human epidermal growth factor receptor 2; ER: estrogen receptor; PgR: progesterone receptor;Triple negative: ER-/PgR-/HER2-
